# Do Past Experience and Group Heterogeneity Matter to Consumer Preferences? Evidence From a Choice Experiment in Urban China

**DOI:** 10.3389/fpsyg.2022.843433

**Published:** 2022-04-27

**Authors:** Ruifeng Liu, Fei Liang, Yan Heng, Zhifeng Gao, Heather Arielle Snell, Allan Rae, Hengyun Ma

**Affiliations:** ^1^College of Economics and Management, Henan Agricultural University, Zhengzhou, China; ^2^Department of Food and Resource Economics, University of Florida, Gainesville, FL, United States; ^3^Department of Agricultural Economics and Agribusiness, University of Arkansas, Fayetteville, AR, United States; ^4^School of Economics and Finance, Massey University, Palmerston North, New Zealand

**Keywords:** past experience, group heterogeneity, choice experiments, consumer preferences, China

## Abstract

This study uses a discrete choice experiment to examine consumers’ preferences for Fuji apple product attributes and willingness to pay (WTP) estimates for consumers in six cities in China. We estimated the preference heterogeneity by linking the stated preference choice data with consumers’ past experience and socioeconomic characteristics in the latent class model. The empirical results show that, first, the past experience variables are crucial in explaining consumer preferences and WTP. Second, three classes, namely, certification-oriented, price- and origin-oriented, and not interested, are identified. Furthermore, the same type of Fuji apple attribute does not appeal to every respondent. Third, our results indicate the heterogeneity of preferences across different classes of respondents, as well as differences in WTP for Fuji apples.

## Introduction

Consumers often make decisions without being sure of their preferences because of bounded rationality, uncertainty, and complexity (Czajkowski et al., 2014; Ding et al., 2020). “Experience goods” means that consumers are not sure of their preferences, but learn their preferences through every consumption event (Nelson, 1970, 1974; Stigler and Becker, 1977). Consumers often have to face complex choice tasks with little prior knowledge but past experience means consumers may find it overwhelming to try to process the information available (Bettman and Park, 1980; Wilcock et al., 2004). Traditionally, research studies on consumer decision making under risk and uncertainty are focused on one-off decision (Lejarraga and Gonzalez, 2011). Such real-world decisions occur repeatedly, but the outcomes of those decisions and their likelihoods often have to be inferred from past experience (e.g., Myers and Sadler, 1960; Edwards, 1961; Katz, 1964). Introspections also show that our preferences and choices often change as we gain experience in a particular market, even if other variables related to choice remain constant (Maltz, 2016).

In addition, preferences are the result of a series of dynamic, experiential choices. As pointed out by Wright and Lynch (1995), direct experience leads to stronger belief, which contributes to higher attitudinal behavioral consistency. As individuals’ choices accumulate, their preferences are constantly updated. When people make choices or purchase decisions about products and services, they can use their principles or experiences to compare attribute levels (Neuman et al., 2010). This kind of experience can improve the decision-making efficiency and reduce transaction cost to some extent (Ding et al., 2020).

The purpose of this study is to investigate the impact of past experiences on respondents’ preferences and WTP for Fuji apples in China. In particular, a Latent Class model (LC model) is used to identify the sources of heterogeneity in preferences across classes of respondents and to evaluate class-specific WTP for the Fuji apple attributes. This study makes two contributions to the existing literature. First, we accounted the sources of heterogeneity including socioeconomic and respondent’s past experiences, and specifically, three past experiences in different contexts are taken into account: (i) consumers used to buy waxed apples; (ii) consumers used to buy apples with pesticide residues; (iii) consumers used to buy counterfeit certified apples. Second, we have empirically examined Chinese consumer preferences for traceable foods, providing timely information on the development of traceability programs in China as an emerging market. As a result, this study will contribute to a better understanding of how past experience and group heterogeneity affect consumers’ preferences and decision making.

The rest of this article is structured as follows. Section “Literature Review” briefly reviews the relevant literature. Section “Data Sources and Sample Description” demonstrates the data and Section “Econometric models” describes the econometric models used here. Section “Results and Analyses” discusses the empirical results. The last section gives the conclusions and implications.

## Literature Review

The interest has rapidly grown in studying the decisions of respondents from past experience. There have been some conceptual analyses ranging from early theories of the impact of different levels of past experience (Howard, 1977), to the processing of consumer memory processes (Olson, 1978). Furthermore, many empirical studies have concluded that people’s preference and willingness to pay (WTP) are influenced by their past experiences (e.g., Ferrini and Scarpa, 2007; Hanley et al., 2009; Ding et al., 2020). For example, Bradbury et al. (2015) found that the simulation experience significantly improved participants’ understanding of potential risk–reward and caused them to reconsider their investment decisions. Kingsley and Brown (2010) noted that as the experience of thinking and comparing accumulated, the respondents became more adept at distinguishing objects. However, Neuman et al. (2010), when studying the effect of experience on health service preference, found that experience does not affect preference.

In addition, there are increasing research studies on how familiarity with markets or valuation mechanisms affect the stated or revealed WTP measures (e.g., List, 2001; Lejarraga and Gonzalez, 2011; Maltz, 2016), while other streams of empirical literature are interested in identifying how learning about goods affects preference uncertainty and consumer demand (e.g., Ackerberg, 2003; Crawford and Shum, 2005; Osborne, 2011). However, currently, there is no widely accepted way to test and control the impact and presence of experience and learning on WTP. This is particularly important for non-market valuation methods, such as conditional valuation and the Discrete Choice Experiment (DCE) (Caputo et al., 2020; Fang et al., 2021; Wang et al., 2022), because the products being valued may not be familiar to many respondents (Bateman et al., 2004; Carson and Louviere, 2011; Czajkowski et al., 2014).

## Data Sources and Sample Description

### Data Source

We use a survey with the choice experiment to examine the effects of consumer experience on their preference and WTP for Fuji apples^1^. From July to October 2017, we conducted face-to-face interviews with urban consumers across six Chinese cities, accounting for the geographical location of different cities, different levels of economic development, and cultural differences. Within each city, we randomly selected four districts according to the administrative divisions. We chose convenient samples to obtain a wide base of Fuji apple respondents at grocery stores, supermarkets, fruit shops, and farmers’ markets across the city.

For this study, respondents were selected as adults (18 years of age or older) who had purchased Fuji apples for the past 6 months. Previous works of literature have found that “*cheap talk*” is an effective way to eliminate hypothetical bias (Lusk, 2003; Silva et al., 2011; Bello and Abdulai, 2016). Because of this, we used a “*cheap talk*” script before conducting the survey (Appendix A). Furthermore, following Loureiro and Umberger (2007) and Savage and Waldman (2008), the choice sets and Fuji apple options in each choice set were randomly sorted to reduce the learning effect and ranking bias of respondents. In our study, the total number of respondents in all cities was 2092, including 408 in Beijing, 413 in Shanghai, 383 in Guangzhou, 324 in Xi’an, 269 in Harbin, and 295 in Jinan.

The attributes chosen for the choice experiment describe four relevant aspects of Fuji apples: traceability information, certification type, region of origin claim, and purchase price. These attributes are the main determinants of apple products in this study. In addition, these attributes have been designed for use in different foods studies such as Meas et al. (2015), Jin et al. (2017), Wongprawmas and Canavari (2017); Caputo et al. (2020), and Alonso et al. (2021). In particular, (i) traceability information is divided into four levels: no traceability information (*NOTRACE*), traceability information that includes only the production part of the value chain (*LOTRACE*), traceability information that includes the production and processing parts of the value chain (*MITRACE*), and traceability information that includes the production, processing, and distribution parts of the value chain (*HITRACE*). (ii) Certification type includes no certification (*NOTHCERT*), government certification (*GOVCERT*), domestic third-party certification (*DOTHCERT*), and international third-party certification (*INTHCERT*). (iii) Region of origin claim consists of four levels: no region of origin claim (*NOCLAIM*), produced in Shandong (*SD*), Xinjiang (*XJ*), and Shaanxi (*SHX*). (iv) Four levels of the purchasing price of Fuji apples are selected: 6 yuan per 500 g, 8 yuan per 500 g, 10 yuan per 500 g, and 12 yuan per 500 g.

Based on the selected Fuji apple product attributes and their levels, a full factorial design generated a total of 256 (4 traceability information × 4 certification types × 4 regions of origin claim × 4 price levels) possible product profiles. Two product profiles pair at random to build a choice set in each selection scenario. As a result, the number of choice sets grew exponentially to 32,640 in this study. As in many choice experiment applications, the number of attributes and their levels are too large for one participant to evaluate all permutations. To reduce the cognitive burden of participants, we used SSIWeb 7.0 software and adopted a randomized design to establish a choice experiment with 120 choice sets^2^. The 120 selection sets are then divided into 10 versions, and respondents are randomly assigned to one of 10 blocks. A ‘‘no-buy’’ option is included in each choice task (Appendix C). A balanced overlap method^3^ minimizing-error for the simulated Random Parameters Logit (RPL)/mixed logit model was employed to generate the choice sets in our study using a seed of 1. We observed that the frequencies of all attribute levels are generally balanced, with the deviation between the actual and ideal standard deviations being less than 10% (except for *HITRACE* and *SD*, see Appendix B).

### Sample Description

Table 1 presents the sample respondents’ characteristics. In our pooled sample, the proportion of men was slightly higher than that of women. The average age of respondents was approximately 34 years. Respondents in this study had about 14.57 years of education. The average monthly income of respondents was approximately 6,410.25 yuan/month, and the monthly family income was 18,210 yuan/month.

**TABLE 1:** Demographic characteristics and past experiences buying Fuji apple of the sample.

Variable	Definition	Mean	Standard deviation
Gender	1 = male; 0 = female	0.507	0.50
Age	Physical age of respondent (years)	34.00	12.75
Education	Educational level of respondent (years)	14.57	3.11
Personal monthly income	Respondent income per capita (yuan/month)	6410.25	14069.65
Family monthly income	Household income per capita (yuan/month)	18210.97	31436.82
Consumer experiences:			
Used to buy waxed apples (self-report)			
*Nb*	1 = I used to buy; 0 = otherwise	0.415	0.49
*Nbm*	1 = uncertain or do not know; 0 = otherwise	0.324	0.47
*no_Nb*	1 = I did not; 0 = otherwise	0.261	0.44
Used to buy apples with pesticide residues (self-report)			
*Np*	1 = I used to buy; 0 = otherwise	0.370	0.48
*Npm*	1 = uncertain or do not know; 0 = otherwise	0.494	0.50
*no_Np*	1 = I did not; 0 = otherwise	0.136	0.34
Used to buy counterfeit certified apples (self-report)			
*Nf*	1 = I used to buy; 0 = otherwise	0.076	0.27
*Nfm*	1 = uncertain or do not know; 0 = otherwise	0.542	0.50
*no_Nf*	1 = I did not; 0 = otherwise	0.382	0.49

Respondents in our study were asked about their experiences of buying waxed apples, apples with pesticide residues, and counterfeit certified apples. When the subjects were asked if they had ever purchased waxed apples, approximately 41.5% of the pooled sample said they had, approximately 32.4% said they were not sure or did not know, and approximately 26.1% said they had not. Furthermore, only 9.49% of respondents in Jinan said they were unsure or did not know if they had ever bought waxed apples. The proportion of respondents in the other five cities answering this question was much higher than that in Jinan.

When the respondents were asked if they had ever purchased apples with pesticide residues, approximately 49.4% of the pooled sample said they were not sure or did not know, approximately 37.1% said they had, and approximately 13.6% said they had not. We also found that the proportion of respondents in Beijing (61.3%), Shanghai (55.9%), and Guangzhou (52.0%) who were unsure or did not know whether they had purchased apples with pesticide residues was higher than in Harbin (42.0%), Xi’an (41.1%), and Jinan (36.3%).

In terms of whether they have ever bought counterfeit certified apples^4^, only 7.6% of respondents self-reported they had, 54.2% of respondents were unsure or did not know, and 38.2% of respondents had not. We also found that the proportion of respondents in each city who answered that question with three options was similar to the proportion in the pooled sample.

## Econometric Models

Based on Lancaster’s (1966) theory of consumer demand, we use an indirect utility function in the modeling of Fuji apple purchasing decisions of consumers. Following random utility theory (McFadden, 1974), consumer *n*′s utility from choosing alternative *j* in a choice situation *t* can be written as:


(1) $$U_{njt}=V_{njt}+\varepsilon_{njt}$$

where *V*_*njt*_ = *X*_*njt*_β. β is a vector of unknown part-worth utilities associated with Fuji apple’s attributes *X*_*njt*_, and ε_*njt*_ is the stochastic and unobserved component of the utility.

When ε_*njt*_ follows a Type I extreme value distribution assuming independently and identically distributed (Maddala, 1997), and the assumption of IIA is valid, a Conditional Logit (CL) model can be employed to estimate the probability of the *j* th option being chosen as:


(2) $$P_{njt}=\frac{\text{exp}\left(X_{njt}\beta \right)}{\sum_{i=1}^{I}\text{exp}\left(X_{nit}\beta \right)}$$

If the assumption of homogeneous preferences is relaxed, then the probability that consumer *n* chooses alternative *j* in choice situation *t* can be written as:


(3) $$P_{njt}=\int \frac{\text{exp}\left(X_{njt}\beta \right)}{\sum_{i=1}^{I}\text{exp}\left(X_{nit}\beta \right)}f\left(\beta \right)d\beta$$

where *f*(⋅) is the distribution of the random preference parameter. If the parameters are fixed at β_*c*_ (non-random), the distribution will collapse, that is, = β_*c*_, then *f*(β_*c*_) = 1. Equation (3) can be estimated as an RPL model, which is an appropriate approach to capturing the heterogeneity in the decision making of consumers (Brownstone and Train, 1999; McFadden and Train, 2000). If *f*(β) is discrete, Eq. (3) can be converted into the Latent Class model (LC model). The probability for consumer *n* falling into class *k* and choosing alternative *j* in choice situation *t* can be expressed as:


(4) $$P_{njt}=\sum_{k=1}^{K}\frac{\text{exp}\left(X_{njt}\beta_{k}\right)}{\sum_{i=1}^{I}\text{exp}\left(X_{nit}\beta_{k}\right)}R_{nk}$$

where β_*k*_ is the parameter vector of the consumer group in class *k*, and *R*_*nk*_ is the probability for consumer *n* falling into class *k*. The corresponding probability can be written as:


(5) $$R_{nk}=\frac{\text{exp}\left(Z_{n}\theta_{k}^{\prime }\right)}{\sum_{r=1}^{R}\text{exp}\left(Z_{r}\theta_{r}^{\prime }\right)}$$

where *Z_n_* is a range of observed values influencing consumer *n* in a certain class, and $\theta_{k}^{\prime }$ denotes the parameter vector of a consumer in class *k*.

Different models are specified in this study: CL Model, RPL Model, and RPL with interactions between attributes. Individual *n*’s utility of choosing alternative *j* in each choice set *t* can be specified as follows:


(6) $$\begin{array}{c} U_{njt}=ASC+\text{P}rice_{njt}\alpha +\text{C}ERTIF{\text{Y}}_{njt}\beta +\text{T}RAC{\text{E}}_{njt}\gamma \\ \;+\text{O}RIGI{\text{N}}_{njt}\delta +\varepsilon_{njt}, \end{array}$$

where *ASC* is an alternative-specific constant denoting the “no-buy” option. **P***rice*_**n***jt*_is a vector of alternative variables represented by the price level of the four experimental designs. ***CERTIFY*** is a vector of alternative certification types of Fuji apples, including government certification (*GOVERT*), domestic third-party certification (*DOTHCERT*), and international third-party certification (*INTHCERT*). ***TRACE*** is a vector of alternative levels of traceability information of Fuji apples, including a high level of tracing information of the value chain (that from production, processing, to distribution parts) (*HITRACE*), a middle level of tracing information (only production and processing parts) (*MITRACE*), and low level of tracing information (only the production part) (*LOTRACE*). ***ORIGIN*** is a vector of alternative region of origin claims of Fuji apples, including Shandong (*SD*), Xinjiang (*XJ*), and Shaanxi (*SHX*). α is the price parameter. β, γ, and δ are parameter vectors measuring respondents’ preferences for non-price attributes. These parameters are assumed to be random, following a normal distribution, while the coefficients of ASC (no-buy variable) and the price are assumed to be fixed as suggested by Ubilava and Foster (2009).

Furthermore, we include the interaction terms of consumers’ past experience in the RPL model. The consumers’ past experiences of buying apples are categorized into three levels: they used to buy a type of apple; they did not know or were unsure about having bought a type of apple; and consumers did not buy the type of apple. The model can be modified as follows:


(7) $$\begin{array}{c} U_{njt}=ASC+\text{P}rice_{njt}\alpha +\text{C}ERTIF{\text{Y}}_{njt}\beta +\text{T}RAC{\text{E}}_{njt}\gamma \\ \;+\text{O}RIGI{\text{N}}_{njt}\delta +\left(\text{E}X{\text{P}}_{n}\times \text{C}ERTIF{\text{Y}}_{njt}\right)\eta \\ \;+\left(\text{E}X{\text{P}}_{n}\times \text{T}RAC{\text{E}}_{njt}\right)\theta +\left(\text{E}X{\text{P}}_{n}\times \text{O}RIGI{\text{N}}_{njt}\right)\sigma \\ \;+\left(\text{U}NSURE_{n}\times \text{C}ERTIF{\text{Y}}_{njt}\right)\lambda +(\text{U}NSUR{\text{E}}_{n}\\ \;\times \text{T}RAC{\text{E}}_{njt})\omega +(\text{U}NSUR{\text{E}}_{n}\times \text{O}RIGI{\text{N}}_{njt})\varphi +\varepsilon {}_{njt} \end{array}$$

where ***EXP*** is a vector of alternative consumers’ past experiences with Fuji apples, including experience of buying waxed apples (*Nb*), the experience of buying apples with pesticide residues (*Np*), and experience of buying counterfeit certified apples (*Nf*). The interaction terms ***EXP*** × ***CERTIFY*, *EXP*** × ***TRACE*,** and ***EXP*** × ***ORIGIN*** represent the interaction effects between consumers’ past experiences of buying apples and their preferences for certification type, traceability information, and the region of origin claim. ***UNSURE*** is a vector of consumers’ uncertainty about their past purchasing of waxed apples (*Nbm*), apples with pesticide residues (*Npm*), and counterfeit certified apples (*Nfm*). The interaction terms of ***UNSURE*** × ***CERTIFY*, *UNSURE*** × ***TRACE*,** and ***UNSURE*** × ***ORIGIN*** represent the interaction effects between consumers’ uncertainty about their past purchases and other attributes. The base of these past experience variables is that respondents did not buy the apples. The definition of consumers’ past experiences in buying Fuji apple is shown in Table 1.

In addition, the WTPs for Fuji apple attributes are calculated by $\text{WTP}=-\frac{\beta_{t}}{\beta_{p}}$, where β_*t*_ is the coefficient of non-price attribute *t*, and β_*p*_ is the estimated price coefficient.

## Results and Analyses

### Results of Conditional Logit Model, Random Parameters Logit Model, and Random Parameters Logit With an Interaction Model

Estimation results of the final specification of the CL, RPL, and RPL with interaction model are presented in this section. Krinsky and Robb (1986) methods are employed to simulate the standard error of coefficients associated with the standard deviation correlation of random parameters. Dummy coding is used for non-price attributes. No traceability information (*NOTRACE*), no certification (*NOTHCERT*), and no region of origin claim (*NOCLAIM*) are set separately as reference levels. All models are estimated using STATA 15.0 and using 1000 Halton draws for the simulation taking into account the panel structure of the data.

Parameter estimates of the main effect variables in the three models are consistent in signs and statistical significance as shown in Table 2. The discussion that follows will be based on RPL with the interaction model, as it reports better data fitting – the maximum value of Log-Likelihood (-21,006.35). Moreover, it allows us to explain the impact of experience on consumer choice and provides richer information about the heterogeneity of consumer tastes. In RPL with the interaction model, the constant for the no-buy option (ASC) and the price coefficient were negative and statistically significant at the 1% level. This means that consumers get less utility from not choosing any of the proposed alternatives than from buying one of them. In addition, increasing the price of Fuji apples reduces consumer utility. The taste parameters of the Fuji apples are all highly significant and in line with the expected sign. There is considerable unobserved heterogeneity of preferences. The results show that respondents perceived certification type as the most important, followed by the region of origin claim and traceability information.

**TABLE 2:** Estimates of parameters for CL, RPL, and RPL with interaction model.

Variables	CL model	RPL model	RPL with interaction model	
	Mean	Mean	Mean	Standard deviation
ASC no-buy	-0.38***(0.05)	–0.49***(0.10)	-0.49***(0.10)	
Price	-0.17***(0.00)	-0.22***(0.10)	-0.22***(0.09)	
Certification type:				
*GOVCERT*	1.17***(0.03)	1.42***(0.05)	1.76***(0.13)	1.16***(0.06)
*DOTHCERT*	0.94***(0.03)	1.16***(0.04)	1.43***(0.12)	0.71***(0.07)
*INTHCERT*	1.06***(0.03)	1.29***(0.05)	1.67***(0.14)	1.04***(0.06)
Traceability information:				
*LOTRACE*	0.41***(0.03)	0.53***(0.04)	0.41***(0.09)	-0.02(0.42)
*MITRACE*	0.63***(0.03)	0.82***(0.04)	0.61***(0.11)	0.73***(0.07)
*HITRACE*	0.83***(0.03)	1.04***(0.05)	0.77***(0.12)	0.80***(0.07)
Region of origin claim:				
*XJ*	0.90***(0.03)	1.10***(0.05)	1.25***(0.12)	0.91***(0.08)
*SD*	0.94***(0.03)	1.15***(0.05)	1.27***(0.13)	0.90***(0.06)
*SHX*	0.93***(0.03)	1.15***(0.04)	1.24***(0.12)	0.87***(0.06)
Interaction term:				
*GOVCERT × Nb*	–	–	0.30**(0.12)	–
*DOTHCERT × Nb*	–	–	0.13(0.11)	–
*INTHCERT × Nb*	–	–	0.11(0.12)	–
*HITRACE × Nb*	–	–	0.19(0.12)	–
*MITRACE × Nb*	–	–	0.10(0.11)	–
*LOTRACE × Nb*	–	–	0.00(0.09)	–
*XJ × Nb*	–	–	–0.17(0.12)	–
*SD × Nb*	–	–	–0.32**(0.13)	–
*SHX × Nb*	–	–	–0.31***(0.12)	–
*GOVCERT × Nbm*	–	–	0.43***(0.13)	–
*DOTHCERT × Nbm*	–	–	0.19*(0.12)	–
*INTHCERT × Nbm*	–	–	0.19(0.12)	–
*HITRACE × Nbm*	–	–	0.12(0.12)	–
*MITRACE × Nbm*	–	–	0.03(0.11)	–
*LOTRACE × Nbm*	–	–	–0.04(0.09)	–
*XJ × Nbm*	–	–	–0.04(0.13)	–
*SD × Nbm*	–	–	–0.40***(0.13)	–
*SHX × Nbm*	–	–	–0.11(0.12)	–
*GOVCERT × Np*	–	–	–0.57***(0.16)	–
*DOTHCERT × Np*	–	–	–0.26*(0.14)	–
*INTHCERT × Np*	–	–	–0.26(0.16)	–
*HITRACE × Np*	–	–	0.04(0.15)	–
*MITRACE × Np*	–	–	0.02(0.14)	–
*LOTRACE × Np*	–	–	0.07(0.11)	–
*XJ × Np*	–	–	–0.01(0.15)	–
*SD × Np*	–	–	0.34**(0.17)	–
*SHX × Np*	–	–	0.25*(0.14)	–
*GOVCERT × Npm*	–	–	–0.71***(0.15)	–
*DOTHCERT × Npm*	–	–	–0.40***(0.14)	–
*INTHCERT × Npm*	–	–	–0.56***(0.15)	–
*HITRACE × Npm*	–	–	–0.01(0.15)	–
*MITRACE × Npm*	–	–	0.01(0.13)	–
*LOTRACE × Npm*	–	–	0.06(0.11)	–
*XJ × Npm*	–	–	–0.05(0.14)	–
*SD × Npm*	–	–	0.24(0.16)	–
*SHX × Npm*	–	–	0.06(0.14)	–
*GOVCERT × Nf*	–	–	0.11(0.19)	–
*DOTHCERT × Nf*	–	–	0.04(0.17)	–
*INTHCERT × Nf*	–	–	0.01(0.18)	–
*HITRACE × Nf*	–	–	0.14(0.15)	–
*MITRACE × Nf*	–	–	0.12(0.16)	–
*LOTRACE × Nf*	–	–	0.25**(0.12)	–
*XJ × Nf*	–	–	0.19(0.19)	–
*SD × Nf*	–	–	–0.16(0.17)	–
*SHX × Nf*	–	–	0.27(0.17)	–
*GOVCERT × Nfm*	–	–	–0.07(0.10)	–
*DOTHCERT × Nfm*	–	–	–0.16*(0.09)	–
*INTHCERT × Nfm*	–	–	–0.20**(0.09)	–
*HITRACE × Nfm*	–	–	0.25***(0.09)	–
*MITRACE × Nfm*	–	–	0.27***(0.09)	–
*LOTRACE × Nfm*	–	–	0.12*(0.07)	–
*XJ × Nfm*	–	–	–0.09(0.09)	–
*SD × Nfm*	–	–	–0.15*(0.09)	–
*SHX × Nfm*	–	–	–0.13(0.09)	–
Respondents	2092	2092	2092	
Observations	75312	75312	75312	
Log Likelihood	–22307.07	–21091.47	-21006.35	

Robust standard errors in parentheses. ***, ** and * indicate significance at 1, 5, and 10% significance levels, respectively.

The main interest of this article is the effect of experience on respondents’ preferences. As regards the interactions of the experience of consumers buying waxed apples interacting with certification type, the coefficient of *GOVCERT × Nb* is positive and significant at the 5% level, while the coefficients of both interaction terms *DOTHCERT × Nb* and *INTHCERT × Nb* are positive and not statistically significant These results indicate that consumers who have bought waxed apples are more inclined to buy Fuji apples with government certification, rather than Fuji apples with international third-party and domestic third-party certification. This also shows that Chinese consumers now trust government certification more, which is consistent with the studies by Bai et al. (2013), Wu et al. (2017), and Liu et al. (2019).

In looking at the interactions of the experience of consumers buying waxed apples interacting with the region of origin claim, the coefficients of both interaction terms *SD × Nb* and *SHX × Nb* are negative and significant, while the coefficient of interaction term *XJ × Nb* is not statistically significant. The results suggest that consumers with the experience of buying waxed apples are not willing to buy Fuji apples from Shandong and Shaanxi provinces. That is, Chinese consumers continue to worry about the safety of waxed Fuji apples and the health risks of excessive consumption, regardless of whether the apples come from Shandong or Shaanxi provinces. In addition, we also find that the interactions of the experience of consumers buying waxed apples interacting with traceability information are not significant.

The coefficient of interaction terms between the experience of consumers who are uncertain or do not know whether they have bought waxed apples with the government certification (*GOVCERT × Nbm*), and with the domestic third-party certification (*DOTHCERT × Nbm*) are positive and significant. The findings indicate that when faced with uncertainty and information asymmetry, the sample subjects are more likely to buy Fuji apples certified by the government and the domestic third party.

We also observe that the coefficient of interaction term *SD × Nbm* is negative and significant. This indicates that when consumers self-report, “they are not sure or do not know whether they have bought waxed apples,” they are not inclined to buy Fuji apples from Shandong Province. This finding has interesting implications for Fuji apple producers and retailers. In other words, if information transparency is increased and although consumers know that the Fuji apples are not waxed, the region of origin labeling will promote consumers to buy Fuji apples from Shandong Province.

Turning to the interactions of the experience of consumers’ self-reported buying apples with pesticide residues interacting with certification type, the coefficients of both interaction terms *GOVCERT × Np* and *DOTHCERT × Np* are negative and significant at the 1 and 5% levels, respectively. These results show that negative experiences of consumers buying apples with pesticide residues decrease their willingness to buy the Fuji apples certified by the government and the domestic third party. The findings are consistent with China’s reality and expectations. In other words, Chinese consumers are concerned about Fuji apple’s pesticide problems, and their past negative experience will weaken the possible positive effect of certification, and ultimately reduce their purchase tendency of Fuji apples. This implies that if Fuji apple producers can produce green or organic apples and minimize consumers’ negative evaluation of pesticide residues in apples, it will effectively increase consumers’ purchase probability of Fuji apples.

The interactions of the experience of consumers buying apples with pesticide residues interacting with a region of origin claim, however, the coefficients of both interaction terms *SD × Np* and *SHX × Np* are positive and significant at 5% and 10% levels, respectively. The findings suggest that consumers who have bought apples with pesticide residues are more likely to buy Fuji apples from Shandong and Shaanxi provinces. The results are interesting, perhaps indicating that consumers have a more positive attitude and trust toward Fuji apples from these two provinces.

In terms of the interactions between the experience of consumers who are uncertain or do not know whether they have bought apples with pesticide residues with the certification type, the coefficients of *GOVCERT × Npm*, *DOTHCERT × Npm*, and *INTHCERT × Npm* are all negative and significant at 1% level. The findings imply that when consumers are uncertain or do not know whether they have bought apples with pesticide residues, they would not tend to buy Fuji apples certified by the government, the domestic third party, and the international third party. The findings are contrary to the above results where the coefficients of *GOVCERT × Nbm* and *DOTHCERT × Nbm* are positive and significant. The possible reason is that consumers may be more concerned about pesticide residues on apples than about apple waxing. In the case of consumer uncertainty or information asymmetry, they will not buy Fuji apples even if they are certified by different certification bodies.

With respect to the interactions of the experience of consumers buying counterfeit certified apples and traceability information, the coefficient of *LOTRACE × Nf* is positive and significant at the 5% level. The result suggests that when consumers self-reported having purchased counterfeit certified apples, they were more likely to buy Fuji apples with traceable information that includes only the production part of the value chain. The findings have important implications for policymakers, Fuji apple producers, and retailers that “more is better” may not be a good strategy for providing traceability information in cases where consumers buy counterfeit certified apples.

In looking at the interactions between the experience of consumers who are uncertain or do not know whether they have bought counterfeit certified apples with the certification type, the coefficients of both interaction terms *DOTHCERT × Nfm* and *INTHCERT × Nfm* are negative and significant at 10 and 5% levels, respectively, while the coefficient of *GOVCERT × Nfm* is not significant. The findings denote that if consumers do not know or are unsure whether they have purchased counterfeit certified apples, this will reduce their propensity to buy Fuji apples certified by domestic and international third parties.

Regarding the interactions between the experience of consumers who are uncertain or do not know whether they have bought counterfeit certified apples with traceability information, the coefficients of *HITRACE × Nfm*, *MITRACE × Nfm*, and *LOTRACE × Nfm* are all positive and significant at 1, 1, and 10% levels, respectively. The results showed that even if consumers have experienced being unsure or not knowing whether they bought a fake certified apple, the traceability information will increase their purchase propensity to Fuji apples. The results are in line with the current situation of Chinese consumers. That is, when Chinese consumers are unsure whether they are buying counterfeit certified apples, even certified Fuji apples, their propensity to purchase is not positive. For policymakers, Fuji apple producers and retailers can increase the supply of traceable information at all levels to improve consumers’ purchase probability of Fuji apple. We also found that the coefficient of *SD × Nfm* is negative and significant at the 10% level, suggesting that the experience of being uncertain or not knowing whether they bought counterfeit certified apples would make them less willing to buy Fuji apples from Shandong Province.

### Willingness to Pay for Attributes of Fuji Apples

Table 3 presents the estimated mean and 95% confidence intervals of consumer WTP for the attributes among the three models. As shown by confidence intervals, WTP values differ significantly in mean and distribution. Based on the results of the three models, the WTP estimates are very stable whether experience variables are or are not included. Furthermore, looking at RPL with the interaction model, adding experience-related interaction terms to the Fuji apples’ study does not significantly decrease the value of WTP estimates (except for the WTP value for traceability information attributes). In general, consumers are willing to pay the highest price for Fuji apples with authenticated attributes, followed by the region of origin claim and traceability information attributes.

**TABLE 3:** Willingness to pay for each attribute level estimated by CL, RPL, and RPL with an interaction model.

Variables	CL model	RPL model	RPL with an interaction model
**Certification type**:			
*GOVCERT*	7.04 [6.59, 7.50]	6.38 [5.75, 7.01]	7.86 [6.58, 9.13]
*DOTHCERT*	5.67 [5.25, 6.09]	5.22 [4.68, 5.76]	6.38 [5.23, 7.53]
*INTHCERT*	6.40 [5.95, 6.85]	5.77 [5.19, 6.36]	7.44 [6.10, 8.78]
**Traceability information:**			
*LOTRACE*	2.46 [2.13, 2.79]	2.39 [2.04, 2.74]	1.81 [0.99, 2.63]
*MITRACE*	3.82 [3.45, 4.20]	3.67 [3.21, 4.13]	2.71 [1.70, 3.71]
*HITRACE*	4.99 [4.58, 5.39]	4.66 [4.14, 5.19]	3.44 [2.32, 4.56]
**Region of origin claim:**			
*XJ*	5.43 [5.00, 6.14]	4.95 [4.41, 5.48]	5.57 [4.46, 6.69]
*SD*	5.70 [5.26, 6.14]	5.14 [4.58, 5.70]	5.65 [4.42, 6.88]
*SHX*	5.63 [5.20, 6.07]	5.15 [4.63, 5.68]	5.52 [4.42, 6.62]
**Interaction term:**			
*GOVCERT × Nb*	–	–	1.36 [0.30, 2.41]
*DOTHCERT × Nb*	–	–	0.56 [–0.38, 1.51]
*INTHCERT × Nb*	–	–	0.50 [–0.54, 1.54]
*HITRACE × Nb*	–	–	0.83 [–0.18, 1.84]
*MITRACE × Nb*	–	–	0.43 [–0.49, 1.34]
*LOTRACE × Nb*	–	–	0.00 [–0.77, 0.77]
*XJ × Nb*	–	–	–0.74 [–1.78, 0.30]
*SD × Nb*	–	–	–1.43 [–2.55, –0.31]
*SHX × Nb*	–	–	–1.37 [–2.39, –0.36]
*GOVCERT × Nbm*	–	–	1.90 [0.75, 3.05]
*DOTHCERT × Nbm*	–	–	0.86 [–0.15, 1.86]
*INTHCERT × Nbm*	–	–	0.85 [–0.24, 1.93]
*HITRACE × Nbm*	–	–	0.54 [–0.53, 1.61]
*MITRACE × Nbm*	–	–	0.14 [–0.83, 1.11]
*LOTRACE × Nbm*	–	–	–0.16 [–0.96, 0.65]
*XJ × Nbm*	–	–	–0.16 [–1.26, 0.94]
*SD × Nbm*	–	–	–1.77 [–2.91, 0.63]
*SHX × Nbm*	–	–	–0.47 [–1.55, 0.61]
*GOVCERT × Np*	–	–	–2.53 [–3.93, 1.13]
*DOTHCERT × Np*	–	–	–1.17 [–2.43, 0.08]
*INTHCERT × Np*	–	–	–1.16 [–2.56, 0.24]
*HITRACE × Np*	–	–	0.19 [–1.11, 1.51]
*MITRACE × Np*	–	–	0.08 [–1.13, 1.29]
*LOTRACE × Np*	–	–	0.33 [–0.65, 1.31]
*XJ × Np*	–	–	–0.03 [–1.34, 1.28]
*SD × Np*	–	–	1.52 [0.08, 2.97]
*SHX × Np*	–	–	1.12 [–0.11, 2.36]
*GOVCERT × Npm*	–	–	–3.17 [–4.51, –1.82]
*DOTHCERT × Npm*	–	–	–1.76 [–2.97, –0.56]
*INTHCERT × Npm*	–	–	–2.51 [–3.87, –1.15]
*HITRACE × Npm*	–	–	–0.03 [–1.31, 1.25]
*MITRACE × Npm*	–	–	0.04 [–1.11, 1.20]
*LOTRACE × Npm*	–	–	0.27 [–0.66, 1.21]
*XJ × Npm*	–	–	–0.21 [–1.47, 1.03]
*SD × Npm*	–	–	1.07 [–0.31, 2.44]
*SHX × Npm*	–	–	0.25 [–0.93, 1.43]
*GOVCERT × Nf*	–	–	0.49 [–1.16, 2.15]
*DOTHCERT × Nf*	–	–	0.20 [–1.27, 1.66]
*INTHCERT × Nf*	–	–	0.06 [–1.51, 1.63]
*HITRACE × Nf*	–	–	0.63 [–0.72, 1.98]
*MITRACE × Nf*	–	–	0.54 [–0.83, 1.91]
*LOTRACE × Nf*	–	–	1.09 [0.05, 2.14]
*XJ × Nf*	–	–	0.86 [–0.83, 2.55]
*SD × Nf*	–	–	–0.69 [–2.18, 0.80]
*SHX × Nf*	–	–	1.21 [–0.29, 2.71]
*GOVCERT × Nfm*	–	–	–0.33 [–1.18, 0.53]
*DOTHCERT × Nfm*	–	–	–0.70 [–1.44, 0.05]
*INTHCERT × Nfm*	–	–	–0.88 [–0.70, –0.07]
*HITRACE × Nfm*	–	–	1.13 [0.31, 1.95]
*MITRACE × Nfm*	–	–	1.19 [0.43, 1.94]
*LOTRACE × Nfm*	–	–	0.54 [–0.07, 1.16]
*XJ × Nfm*	–	–	–0.42 [–1.23, 0.40]
*SD × Nfm*	–	–	–0.69 [–1.49, 0.12]
*SHX × Nfm*	–	–	–0.56 [–1.37, 0.26]

*Numbers in brackets represent 95% confidence intervals, which are estimated by using the parametric bootstrapping procedure of Krinsky and Robb (1986).*

Regarding certification type, we see that the premiums are 7.86 yuan per 500 g for the Fuji apples certified by the government, followed by the international third-party (7.44 yuan per 500 g) and the domestic third-party certification (6.38 yuan per 500 g) compared with Fuji apples with no certification. The WTP results with respect to the certification attributes are expected, given the current situation in China as discussed above. That is, consumers are more likely to trust the government. It is worth noting that consumers do not pay a higher premium for Fuji apples with traceability information compared to certification type and regions of origin claim attributes. In particular, we find that Fuji apples carrying traceability information that includes production, processing, and distribution parts of the value chain would receive a premium of 3.44 yuan per 500 g more than the counterpart carrying no traceability information. A possible explanation for this is that consumers are unfamiliar with traceability and traceable foods and are less willing to pay too much for traceability information attributes. To some extent, this also shows that the current construction and promotion of the food traceability system in China has not met the expectations.

### Consumers’ Class Preference and Willingness to Pay

We identified the latent consumer segments and determined the source of heterogeneity by their socio-demographic and experience variables. We benchmarked model specification searches for conditional Logit specifications with fixed utility coefficients, where all respondents were constrained under the hypothesis of ‘‘preference cloning.’’ Then we ran a canonical search to explore the dimensions of preference heterogeneity between 2 and 10 preference classes^5^. The minimum Akaike information criterion (AIC) and the minimum Bayesian information criterion (BIC) (Allenby, 1990) are used to identify the optimal number of latent preference classes to fit the data. Following Boxall and Adamowicz (2002), the best model is selected according to the two comprehensive criteria of the credibility of the parameter estimation and the leveling of the marginal improvement of the AIC and BIC values as a new class is added. This combined method indicates that the three preference-class models are best. In a nutshell, comfortingly, the latent preference classes are divided into groups of consumers’ choices of Fuji apple have obvious propensities in this study.

Table 4 presents the estimated parameters of the three-class model. With regard to membership probabilities of preference classes, respondents suggest a 65.6% probability of belonging to Class 1, 19.4% belonging to Class 2, and 15.0% to Class 3. The estimated results for the membership equation variables in Class 1 and Class 2 are associated with those in Class 3, which serves as a baseline and thus includes no scores. The sample consumers’ age, education, family income, and experiences of uncertain whether they have bought apples with pesticide residues (*nbn_pesti*) and counterfeit certified apples (*nb_fak*) are all found to be useful to determine class membership. In contrast, other consumers’ experience of buying waxed apples (*nb_wax*), buying apples with pesticide residues (*nb_pesti*), and being uncertain or do not know whether they have bought waxed apples (*nbn_wax*) and counterfeit certified apples (*nbn_fak*) are all not helpful for predicting class membership.

**TABLE 4:** Estimates from latent class model (LC model).

Variable	Class 1 (Certification -oriented)	Class 2 (Price and origin-oriented)	Class 3 (Not interested)
**Class size**	0.656	0.194	0.150
**Attributes:**			
*Price*	-0.09*** (0.01)	-0.12*** (0.02)	-0.71*** (0.04)
*Chooseno*	0.99*** (0.10)	2.41*** (0.26)	4.89*** (0.41)
**Certification type:**			
*GOVCERT*	1.40*** (0.04)	1.22*** (0.12)	0.66*** (0.12)
*DOTHCERT*	1.13*** (0.04)	0.80*** (0.12)	0.55*** (0.10)
*INTHCERT*	1.29*** (0.04)	0.89*** (0.12)	0.76*** (0.10)
**Traceability information:**			
*LOTRACE*	0.51*** (0.03)	0.33*** (0.11)	0.26** (0.10)
*MITRACE*	0.82*** (0.04)	0.61*** (0.11)	0.22** (0.11)
*HITRACE*	1.02*** (0.04)	0.94*** (0.11)	0.39*** (0.11)
**Region of origin claim:**			
*XJ*	1.06*** (0.04)	1.11*** (0.13)	1.08*** (0.10)
*SD*	1.10*** (0.04)	1.31*** (0.12)	1.00*** (0.10)
*SHX*	1.01*** (0.04)	1.05*** (0.13)	1.44*** (0.11)
**Membership Equations:**			
*Age*	-0.02***(0.00)	-0.02*** (0.01)	–
*Education*	0.07*** (0.02)	0.02(0.02)	–
*Family income*	0.00** (0.00)	0.00** (0.00)	–
*nb_wax*	-0.03 (0.18)	0.18 (0.29)	–
*nbn_wax*	-0.12 (0.18)	0.11 (0.28)	–
*nb_pesti*	-0.18 (0.23)	-0.11 (0.45)	–
*nbn_pesti*	-0.40* (0.20)	-0.05 (0.38)	–
*nb_fak*	0.70** (0.31)	0.09 (0.42)	–
*nbn_fak*	–0.06(0.14)	0.06 (0.19)	
*Constant*	1.89*** (0.26)	–0.30 (0.00)	–
Observations	75,312	75,312	75,312
No. of groups	25,104	25,104	25,104

Standard errors in parentheses. *, **, and *** indicate significance at 10, 5, and 1% significance levels, respectively.

The certification type is observed to be of the highest importance for respondents’ Fuji apple choice when compared to traceability information and region of origin claim. Therefore, Class 1 is named as “*Certification-oriented.*” Respondents in Class 1 are more likely to choose lower-priced over higher-priced Fuji apple. This finding is consistent with most studies that price negatively affects purchasing decisions. It indicates that a price increase reduces the demand for Fuji apples. By contrast, this result does not support the findings of Rao (2005) and El Benni et al. (2019). They found that higher prices can be indicators of higher quality products. Qiao et al. (2012) also suggested that buying expensive products reflects the Chinese perception that cheap products are inferior. In addition, compared to Class 3, respondents in Class 1 are likely to be younger, have a higher monthly family income, have higher education, and have no experience or not knowing whether they have bought apples with pesticide residues, but have experience of buying counterfeit apples.

Respondents in Class 2 preferred price and region of origin claim to those in Class 3 in this study. This indicates that Fuji apples related to the declaration of origin are significantly more likely to be selected by Class 2 respondents. In addition, an important feature of Class 2 is the high value of the price parameter, indicating higher price sensitivity compared to other classes. Therefore, we named Class 2 “*Price and origin-oriented.*” The results have important implications for fresh fruit producers in China, that is, that protection and differentiation of region and origin may be important tools for influencing consumer decisions. The findings are in line with most studies such as Wu et al. (2017) and Liu et al. (2020). In addition, respondents in Class 2 are likely to be younger and have a higher monthly family income than those in Class 3.

The coefficient of “*Chooseno*” variable in Class 3 is positive significantly, the value of which is greater than the other attributes of Fuji apples, implying that respondents in Class 3 preferences tend not to choose any other attributes. Therefore, we named Class 3 “*Not interested.*”

Table 5 reports the marginal WTP estimates for each latent class. The results showed that there are significant differences in the marginal WTP among different attributes and consumer classes for Fuji apples. For example, respondents in Class 1 are willing to pay a premium of 12.67 yuan per 500 g Fuji apples from Shandong Province. By contrast, the marginal WTPs of respondents in Class 2 and Class 3 are 10.08 yuan per 500 g and 1.41 yuan per 500 g, respectively.

**TABLE 5:** Marginal WTP estimates for each latent class.

Variable	Class1 (Certification -oriented)	Class 2 (Price and origin-oriented)	Class 3 (Not interested)
**Certification type:**			
*GOVCERT*	16.14 [13.36, 18.92]	7.93 [4.85, 11.01]	0.93 [0.56, 1.30]
*DOTHCERT*	13.10 [10.79, 15.40]	6.78 [3.98, 9.58]	0.77 [0.46, 1.08]
*INTHCERT*	14.92 [12.29, 17.54]	7.58 [4.64, 10.52]	1.08 [0.77, 1.38]
**Traceability information:**			
*LOTRACE*	5.90 [4.71, 7.09]	2.80 [0.82, 4.79]	0.36 [0.07, 0.65]
*MITRACE*	9.49 [7.72, 11.25]	5.13 [2.68, 7.59]	0.31 [-0.01, 0.63]
*HITRACE*	11.80 [9.71, 13.89]	7.69 [4.51, 10.87]	0.55 [0.21, 0.90]
**Region of origin claim:**			
*XJ*	12.22 [10.04, 14.41]	9.41 [5.61, 13.20]	1.52 [1.18, 1.87]
*SD*	12.67 [10.44, 14.90]	10.08 [6.89, 15.28]	1.41 [1.07, 1.75]
*SHX*	11.63 [9.60, 13.67]	8.87 [5.25, 12.49]	2.04 [1.65, 2.43]

*Numbers in brackets represent 95% confidence intervals, which are estimated by using the parametric bootstrapping procedure of Krinsky and Robb (1986).*

## Conclusion and Implications

This study focused on the impact of past purchase experience on consumers’ preferences for Fuji apples using a discrete choice experiment approach. Meanwhile, this study also employs an LC model to investigate the sources of heterogeneity in the preferences of respondents in different classes and to estimate the WTP of Fuji apple attributes in different categories.

### Conclusion

The estimated results show that the most preferred Fuji apple attributes of respondents are the certification type, followed by the region of origin claim and traceability information. For example, in terms of certification type, respondents value Fuji apple certified by the government the most, and those certified by the domestic third party the least. The results also reveal profound heterogeneity in preferences relating to respondents’ past purchase experience and socioeconomic characteristics. Three different consumer classes are identified in the sample population, each showing a different preference for the same set of Fuji apple attributes. In particular, certification type is found the most preferred attribute for “*Certification-oriented”* consumer group, price and region of origin claim are found the most important attributes for “*Price” and “origin-oriented”* consumer group, tending not to choose any other attributes is preferred the most for “*Not interested”* consumer group. In addition, the estimates of WTP also change across different consumer classes.

These findings suggest that past experience plays an important role in explaining class membership and consumer choice behavior. In detail, the coefficients of the interaction terms between experiences and attributes show large differences. This indicates that different past experiences have different effects on respondents’ attribute preferences.

First, if respondents had purchased waxed apples, they are more likely to purchase Fuji apples certified by the government, but less likely to purchase Fuji apples from Shandong and Shaanxi provinces. In contrast, if respondents did not know whether they had ever bought waxed apples, they would reduce buying Fuji apples certified by the government and the domestic third party from Shandong Province.

Second, if respondents had purchased apples with pesticide residues, they are less likely to purchase Fuji apples certified by the government and the domestic third party, and more inclined to buy Fuji apples from Shandong and Shaanxi provinces. If respondents did not know or were unsure whether they had purchased apples containing pesticide residues, they would reduce buying Fuji apples certified by either party.

Third, if respondents had purchased counterfeit certified apples, they are more likely to buy Fuji apples with traceability information including only the production part of the value chain. In addition, we found that if respondents did not know or were unsure whether they had purchased counterfeit certified apples, they are less likely to purchase Fuji apples certified by the domestic third party and international third party, as well as from Shandong Province.

### Implications

Our findings have several important implications for promotion and marketing strategies. First, since consumer past experiences are pervasive, they are crucial to explaining consumer preferences and choice behavior. As a result, ignoring consumers’ past experience would make the estimates of consumer preferences and choice behavior biased. Therefore, the impacts of consumers’ past experiences on their preferences and WTP should be paid attention theoretically and practically.

Second, marketers can tailor their marketing strategies to consumers’ different types of past experiences. For example, consumers who have bought waxed apples, or do not know if they have, are more likely to buy government-certified Fuji apples because of the higher utility they can obtain from them. Therefore, government certification for Fuji apples may be an effective strategy for producers. We understand from this study that if consumers do not know whether they have bought waxed apples, apples with pesticide residues, and counterfeit certified apples, they will be less likely to buy Fuji apples. Therefore, providing Fuji apple attribute information can not only reduce information asymmetry but also improve consumers’ cognition and purchase possibility. On the other hand, it may be necessary to help consumers weigh the information generated when they compare Fuji apple attributes.

Third, our results further indicate that each consumer has his preferences for the same type of attributes. In particular, Fuji apple producers can consider customizing labels to the needs of specific consumer groups to make their marketing strategies effective. It is worth noting that when consumers are faced with multi-attribute integrated labels, they may not be able to learn the information conveyed by the labels. Especially for consumer groups with the least knowledge and experience, marketers or public policymakers may need to make sure that inexperienced consumers will not easily give up buying Fuji apple products.

However, this study still has some limitations. First, the study did not take into account other attributes of Fuji apples, such as color, freshness, taste, brand, and labeling. However, studies have shown that these attributes also influence consumers’ preference and WTP for fresh products (e.g., Wang and Huo, 2016; He et al., 2020, 2021; DeLong et al., 2021; Tanemura and Hamadate, 2022; Wang et al., 2022). Second, our sample was limited to consumers only in six cities in China, so a future study may cover a wider range of urban consumers nationwide to conduct more robust tests.

## Data Availability Statement

The original contributions presented in the study are included in the article/Supplementary Material, further inquiries can be directed to the corresponding author/s.

## Author Contributions

RL: contributes to making draft and revising. FL: conducts field survey and data processing. YH: help question consulting. ZG: revises and reviews. HS: runs statistical software. AR: edits and revises. HM: performs reviewing, editing, and revising as well as corresponding author. All authors contributed to the article and approved the submitted version.

## Conflict of Interest

The authors declare that the research was conducted in the absence of any commercial or financial relationships that could be construed as a potential conflict of interest.

## Publisher’s Note

All claims expressed in this article are solely those of the authors and do not necessarily represent those of their affiliated organizations, or those of the publisher, the editors and the reviewers. Any product that may be evaluated in this article, or claim that may be made by its manufacturer, is not guaranteed or endorsed by the publisher.

## Footnotes

1 The reason to choose Fuji apple as research object is because apple is one of the most common and popular fruit that is consumed nationwide and year-around, and Fuji apple is one of the most favorite among eight major apple varieties in China. According to the 2021–2027 China apple industry market competition situation and market demand potential report by Zhiyan Consulting, National Bureau of Statistics, total fruit output is 286.924 million metric tons, while total apple output is 44.066 million tons, accounting for 15.36% of total fruit output in 2020 in China, refer to: https://www.chyxx.com/research/202010/902605.html.

2 The final choice experiment D-efficiency statistics are summarized in Appendix B. The priori estimates of standard errors for attribute levels are also provided by SSIWeb 7.0. Ideal standard deviation is the standard deviation that meets the orthogonality condition. The “strength” of design for this model is 2,697.15. The ratio of “strengths” of design for two designs reflects the D-efficiency of one design relative to the other.

3 Overlap provides a method to simplify multiple-choice questions, reducing the number of attribute differences that respondents must evaluate (Johnson et al., 2013), and thus is statistically valid (Trenz, 2015).

4 Consumers reported and themselves thought they had once brought counterfeit certified apples.

5 A comparison of LC model with different number of classes is available upon request.
