# Supplementary material for: Do Past Experience and Group Heterogeneity Matter to Consumer Preferences? Evidence From a Choice Experiment in Urban China
Source: Front Psychol. 2022 Apr 27;13:843433. doi: 10.3389/fpsyg.2022.843433 (PMC9095496; doi:10.3389/fpsyg.2022.843433)
Supplement: Supplementary file 1 [file Data_Sheet_1.pdf]

Appendix A. “*cheap talk*” script

“From previous studies we know that people often respond in one way but act differently. People were asked whether they would buy a new product similar to the one you are about to be asked about. Several people stated a higher willingness to pay than what one actually was willing to pay for the product in a grocery store. No one actually had to pay money when the indicated a particular preference. A possible reason for this is that people do not really take into account the limited amount of money we have. It is easy to be generous when you do not really have to pay for it. But when we are really in the grocery store, and we would actually have to spend our money if we decide to buy the product, we may think a different way: the amount of money spent on this product cannot be spent to other things. In any case, we ask you to respond to each of the following preference and willingness to pay questions just exactly as you would if you were in a real grocery store and had to pay for your choice. Please keep this in mind when answering the last few questions.”

Appendix B.

A priori estimates of standard errors for attribute levels

Attribute	Level	Frequency	Actual standard deviation	Ideal standard deviation	Efficiency
Traceability information	*LOTRACE*	60	0.2270	0.2346	1.0679
	MITRACE	60	0.2293	0.2346	1.0468
	HITRACE	60	0.2496	0.2346	0.8839
	NOTRACE	60	-	-	-
Certification type	*GOVCERT*	61	0.2277	0.2325	1.0426
	DOTHCERT	59	0.2391	0.2325	0.9458
	INTHCERT	60	0.2341	0.2325	0.9865
	NOCERT	60	-	-	-
Region of origin claim	*SD*	61	0.2456	0.2325	0.8958
	XJ	60	0.2386	0.2325	0.9496
	SHX	60	0.2341	0.2325	0.9862
	NOORIGIN	59	-	-	-
Price	12 yuan	59	0.2224	0.2294	1.0645
	10 yuan	60	0.2411	0.2294	0.9055
	8 yuan	60	0.2229	0.2294	1.0593
	6 yuan	61	-	-	-

Appendix C. Example of a choice set included in the choice experiment

**Option A**	**Option B**	**Option C**
Traceability information that only includes the production of the value chain	No traceability information	I will not buy
No certification	No certification	
Xinjiang	No region of origin claim	
Price：8 yuan per 500 g	Price：6 yuan per 500 g	
◎	◎	◎
I choose…		
